# Correction to “Fibrinogen‐Derived Fibrinostatin Inhibits Tumorgrowth Through Anti‐Angiogenesis”

**DOI:** 10.1111/cas.70391

**Published:** 2026-04-09

Zhao C, Su Y, Zhang J, Feng Q, Qu L, Wang L, Liu C, Jiang B, Meng L, Shou C. Fibrinogen‐derived fibrinostatin inhibits tumor growth through anti‐angiogenesis. *Cancer Science*. 2015; 106:1596–1606. https://doi.org/10.1111/cas.12797.

In the above article, Figure 6 was incorrect. The correct image for Figure 6 is shown below:

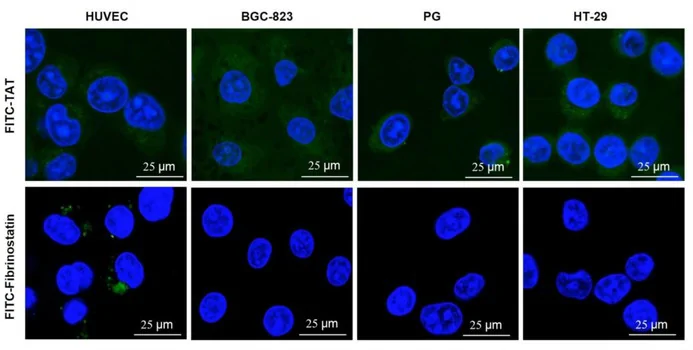

We apologize for this error.

